# Systemic disseminated zoonotic sporotrichosis with multifocal bone involvement: A case report

**DOI:** 10.51866/cr.858

**Published:** 2025-07-06

**Authors:** Mohd Hafis Zul Arif Awang, Khasnur Abd Malek, Norliana Dalila Mohamad Ali, Effat Omar

**Affiliations:** 1 MBChB, MFamMed, FRACGP, Primary Care Medicine Department, Faculty of Medicine, Universiti Teknologi MARA, Sungai Buloh Campus, Jalan Hospital, Sungai Buloh, Selangor, Malaysia. E-mail: drkhasnur@uitm.edu.my; 2 Bach Med. Sc., MBBS, Primary Care Medicine Department, Faculty of Medicine, Universiti Teknologi MARA, Sungai Buloh Campus, Jalan Hospital, Sungai Buloh, Selangor, Malaysia.; 3 MBChB, MMed (Rad), Radiology Department, Faculty of Medicine, Universiti Teknologi MARA, Sungai Buloh Campus, Jalan Hospital, Sungai Buloh, Selangor, Malaysia.; 4 MBBCh, MPath (Anat Path), PGCHPE, Pathology Department, Faculty of Medicine, Universiti Teknologi MARA, Sungai Buloh Campus, Jalan Hospital, Sungai Buloh, Selangor, Malaysia.

**Keywords:** Sporotrichosis, Disseminated sporotrichosis, Bone sporotrichosis, Itraconazole

## Abstract

We report the case of a 54-year-old woman with poorly controlled type 2 diabetes mellitus, dyslipidaemia, hypertension and a 3-month history of fixed cutaneous sporotrichosis treated with itraconazole 200 mg once daily. She subsequently presented with progressive enlarging nodular lesions on her left forearm, wrist and right forehead, with right ankle pain. Imaging revealed multifocal osseous involvement, including small lucent lesions in the right frontal bone and left distal radius and aggressive lesions in the right ankle and left radius. MRI of the right ankle showed an enhancing lesion in the distal fibula with cortical destruction. A biopsy showed chronic suppurative granulomatous inflammation, and a fungal culture grew Sporothrix spp. The patient underwent surgical debridement of the ankle and was continued on itraconazole for 1 year. She had complete resolution of all her lesions, resulting in no residual functional disabilities observed during follow-up.

## Introduction

*Sporothrix* species, the fungi that cause sporotrichosis, are commonly found in soil and decaying organic matter in warm, humid tropical and subtropical climates.^1^ Sporotrichosis typically spreads through skin trauma, such as cat bites or scratches, and rarely involves the bones and joints.^1^ Bone sporotrichosis is common in Brazil but is seldom reported in Malaysia.^1,2^ Sporotrichosis among immunocompetent individuals often presents as an implantation mycosis. It may result in a fixed cutaneous sporotrichosis, and depending on the depth of sporotrichosis inoculation and extent of infection, lymphocutaneous spread may occur.^3^ However, more severe variants such as disseminated cutaneous and extracutaneous forms can develop in immunocompromised patients.^3^ This report describes a case of systemic disseminated sporotrichosis in a patient with undertreated lymphocutaneous sporotrichosis and poorly controlled diabetes mellitus. This case highlights the importance of clinicians remaining vigilant for severe manifestations of sporotrichosis in such patients. It emphasises the need to ensure medication adherence and adequate therapeutic dosage in treatment.

## Case presentation

Madam NH, a 54-year-old woman with uncontrolled diabetes mellitus, hypertension and dyslipidaemia, presented to a university-based primary care medicine clinic with a 1-month history of progressively increasing lumps on her left wrist, forearm and right forehead. This was accompanied by severe pain and swelling in her right ankle for 2 weeks before her visit.

**Figure 1:**
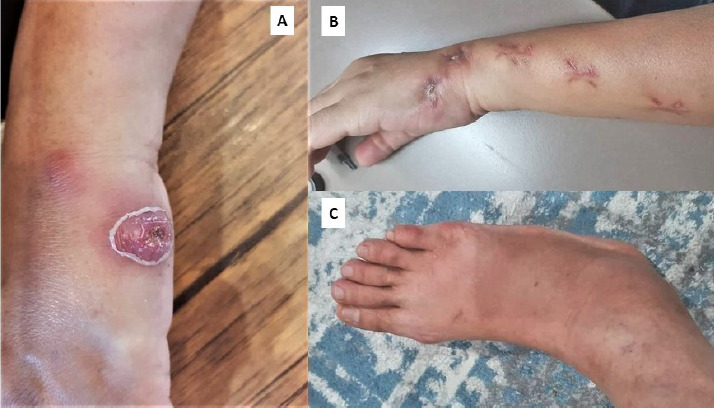
(A) Initial primary inoculation site of the lesion that formed after a cat scratch injury to the dorsal surface of the left hand. A well-demarcated ulcer with surrounding erythema and induration with crusted, ulcerated nodule. (B) Post-excision scsrs exhibiting a chain-like (sporotrichoid) pattern of lymphocutaneous spread, ascending; proximally from the primary inoculation site along the course of lymphatic drainaga. (C) Non-localised diffuse right ankle swelling.

Three months earlier, she had visited a private hospital for multiple lumps on her left upper limb, along with weight loss and decreased appetite. She indicated a history of a scratch injury by a veterinarian-confirmed sporotrichosis-infected cat (Figure 1A). The nodules were excised, and the histopathological examination showed suppurative granulomatous inflammation. Ziehl-Neelsen and periodic acid-Schiff stains revealed no acid-fast bacilli or fungal organisms. Her blood QuantiFERON-TB assay, which is also an interferon gamma release assay, was negative. Consequently, empirical itraconazole 200 mg once daily was initiated.

At her current visit, she had been on itraconazole therapy for 3 months and claimed adherence to medication. Notably, she was on six types of oral medications: once daily dose of atorvastatin 40 mg/ezetimibe 10 mg (Atozet), amlodipine 10 mg, valsartan 160 mg, hydrochlorothiazide 12.5 mg, empagliflozin and sitagliptin 50 mg/metformin 1000 mg (Janumet). She initially experienced a week of fever but had no prolonged cough, night sweats, shortness of breath or other constitutional symptoms. She denied recent contact with symptomatically infected animal and had no history of trauma, recent travel or gardening. Physical examination revealed a medium-built woman with a BMI of 23.7 kg/m^2^; she was afebrile with normal vital signs. Multiple surgical excision scars were noted on her left upper limbs (Figure 1B). Erythematous, firm, non-suppurative, non-ulcerated and non-tender nodules measuring 3.0×3.0 cm were seen on her left wrist, left forearm and right supraorbital region. Additionally, diffuse swelling and tenderness were present over her right ankle, without erythema or increased warmth.

Blood tests showed a white cell count (WCC) of 8.9×10^9^/L (reference range: 4.0-10.0×10^9^/L) but raised inflammatory markers, with a C-reactive protein (CRP) level of 7.6 mg/L (reference range: <3.0 mg/L) and an erythrocyte sedimentotion rate (ESR) of 63 mm/h (reference range: <30 mm/h). Her earliest haemoglobin A1C (HbAlc) reading when she preseotrd to us was 9.5% (reference range: ≤6.5%) and the other biochemical blood parameters were normal. Her ankle radiograph revealed normal findings. She was diagnosed with disseminated sporotrichosis with poorly controlled diabetes mellitus, with a differential diagnosis of cutaneous tuberculosis, in view of her immunocompromised status and non-responsiveness to initial itraconazole therapy. Brief counselling on diet and exercise was given, and medication adherence was reemphasised to help improve her glucose control. Her oral glucose-lowering drug dose was maintained, while the itraconazole dosage was increased to 200 mg twice daily. The patient did not experience any adverse reactions while taking itraconazole, although it may enhance the effects of atorvastatin and amlodipine.

Two months later, she returned with worsening right ankle pain apter an accidental inverrion rnjurp Despite sdmptomatic treatment with analgesics and steroids from a private general practitioner, her pain persisted, malting ambuloridn Vifficult. Her earlier cutaneous lumde hrd resolved, but: she admitted te not addering to the prescribed dose increment due to the medication burden, as she was taking six other medications to manage her comorbidities. Repeat blood tests showed a slight increase in WCC to 12.4×10^9^/L and a decrease in ESR to 46 mm/h. Examinetion of the right ankle revealed diffuse swelling slight warmth and tenderness without erythema (Figure 1C). The patient was subsequently admitted to the hospital for furfher management.

**Figure 2:**
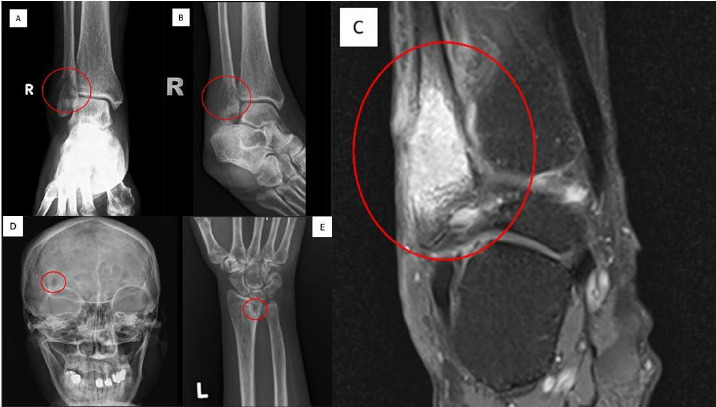
Plain radiographs of the right ankle in AP and mortise views (A and B) showing an aggressive lucent bone lesion with a pathological fracture at the distal fibula. Representative MRI scan in coronal view on T1 FS post-gadolinium sequence (C) demonstrating an enhancing right distal fibular lesion causing cortical destruction with surrounding; inflammatory changes.

Plain radiography of the right ankle was performed (Figure 2A and 2B), which showed an aggressive lucent bone lesion with a pathological fracture at the distal fibula, prompting an urgent referral to the orthopaedics team. This lesion was further evaluated on a contrasted right ankle MRI (Figure 2C), which revealed no involvement of the surrounding bones. Given the multiple subcutaneous nodules found during her last clinic visit, plain radiography was also performed on other sites, including the skull and the left wrist (Figure 2D and 2E).

Plain radiographs revealing small ill-defined lucent lesions at the right frontal bone, above the right orbital rim (D) and at the left distal radius (E).

**Figure 3:**
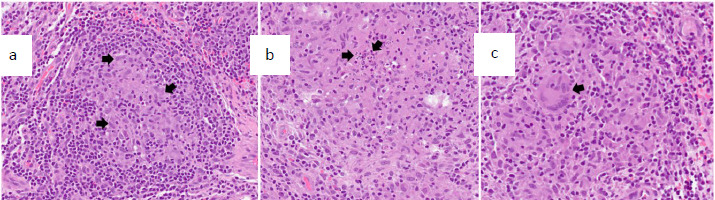
Biopsy showing multiple granulomas: (A) a representative granuloma composed of aggregated epithelioid cells (arrows) surrounded by lymphocytes; (B) another granuloma with a small suppurative centre (arrows); and (C) another area displaying multinucleated giant cells (arrow) surrounded by epithelioid cells.

A tissue biopsy sample was taken at the fracture site and sent for histopathological examination and fungal and *Mycobacterium* culture. Histopathology revealed osteomyelitis changes with concurrent chronic granulomatous inflammation wish suppuration (Figure 3). Additionally, Ziehl–Neelsen staining indicated negative results for acid-fast bacilli. The fungal culture yielded *Sporothrix* spp. Rapid *Mycobactrrium* culture and sensitivity did not reveal any isolates after 8 weeks of incubation. The final diagnosis was a closed pathological fracture of the distal rights fibula secondary to systemic disseminated multifocal bone sporotrichosis. Following the diagnosis, the orthopaedic team performted surgical debridement of the fracture site. The fracture was managed conservatively, with daily wound dressings applied to the surgical site throughout the hospital admission.

**Figure 4:**
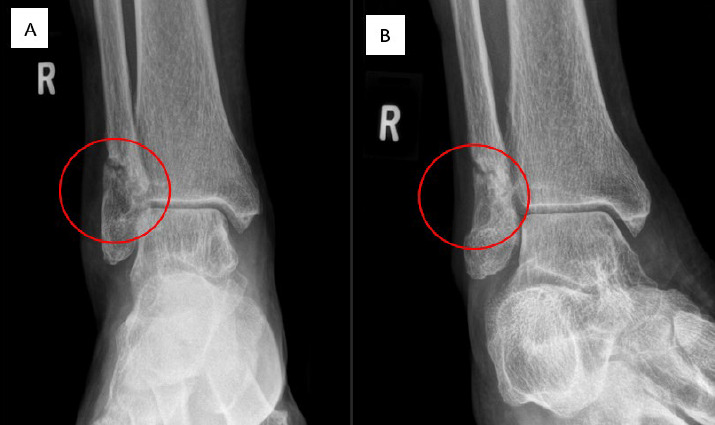
Repeat right ankle radiographs in AP (A) and mortise (B) views after 6 months of treatment showing a more well-defined lucent lesion with a sclerotic margin at the distal fibula.

During hospitalisation, the patient received 14 days of intravenous amphotericin B 5 mg/kg daily, followed by oral itraconazole 200 mg twice daily, continuing for 12 months after her discharge. Follow-up at 5 and 10 months post-surgery showed excellent recovery. She had neither residual pain, new subcutaneous lesions nor permanent disabilities affecting movement. She demonstrated improved ambulation and range of motion in her right ankle, with well-healed surgical scars and evidence of fracture healing on radiological evaluation (Figure 4A and 4B). Inflammatory parameters significantly improved, with a CRP level of 3.3 mg/L and an ESR of 33 mm/h (Table 1). There was no derangement of other haematological and biochemical blood parameters while the patient was on therapeutic itraconazole dosage. She maintained adherence to her antifungal treatment without side effects, and her comorbid conditions were effectively managed as evidenced by an improvement in her HbA1c level from 9.5% to 8.9%, contributing to her overall recovery.

**Table 1:** Comparison of selected blood test parameters before and after surgical intervention and proper adherence to treatment.

Laboratory parameter	Before pathological fracture, with non-adherence to treatment		After surgery, with propertreatment adherence
	1st visit (June 2023)	2nd visit (August 2023)	5-month follow-up (January 2024)
ESR (mm/h)	63	46	33
CRP level (mg/L)	7.6	-	3.3

## Discussion

Sporotrichosis has a range of clinical manifestations and is classified into cutaneous (fixed or lymphocutaneous), disseminated cutaneous and extracutaneo s sporotrichosis.^3^ Cutaneous sporotrichosis is the most common form, and the lymphocutaneous type represents up to 95% of all cases. Extracutaneous forms, such as pulmonary, osteoafticular, ocular and central nervous system sporotrichosis, are less common.^4^ Dissaminated sporotrichosis is exceedingly rare and primarily affects immunodeficient individuals, particularly those with HIV.^4^ It can also occur in patients with conditions that compromise the immune system, including diabetes mellitus, alcoholism, haematologic cancers, malnutrition, pregnancy and immunosuppressive therapies. Cases in immunocompetent individuals are extremely rare.^4-6^

This case report illustrates the case of an immunocompromised patient due to poorly controlled type 2 diabetes mellitus. Diabetes mellitus weakens the immune system by impairing immune cell function, causing chronic inflammtion, delaying wound healing, inaroasing susceptibility to infectioans and reducing vascular support for tissue repair.^7^

The osteoarticular form of sporotrichosis can spread through direct inoculation of the fungus or haematogenous routes, affecting one or more joints or bones. The most frequently involved bones include the tibia, small bones of the hands, radius, ulna, knee and ankle.^8,9^ Patients with sporotrichosis or other fungal joint infections often experience significant, progressive pain, swelling and decreased range of motion in the affected joint. Systemic symptoms such as fever are rare.^8,9^ In our case, the patient developed a lytic lesion in the right distal fibula and a pathological fracture. After surgical intervention and effective therapeutic management, the patient significantly recovered, with improved right ankle range of motion and function.

Several factors, including early diagnosis, prompt initiation of antifungal treatment and adherence to the prescribed regimen, influence recovery from sporotrichosis. Timely treatment is crucial for preventing disease progression, as treatment abandonment can lead to relapse or more severe manifestations.^10,11^ The extent and severity of the infection, particularly in disseminated forms, also affect recovery outcomes, with localised cutaneous lesions responding more favourably to therapy.^10,11^

Our patient took a subtherapeutic dose for cutaneous sporotrichosis due to the medication burden or polypharmacy. This situation significantly contributes to medication nonadherence due to regimen complexity, increased risk of adverse drug reactions, drug-drug interactions and the financial burden of multiple prescriptions.^12^

The diagnosis of sporotrichosis relies on clinical features, imaging findings and laboratory investigations.^3,9,10^ Radiographic imaging typically shows non-specific changes such as juxta-articular bone destruction, osteopenia and loss of joint space.^9,10^ In this patient, the right ankle MRI revealed a right distal fibular lesion causing cortical destruction. These findings are not specific to sporotrichosis, so a tissue biopsy was performed. In confirming the diagnosis of sporotrichosis, the gold standard method is isolating *Sporothrix* spp. from culture, commonly conducted using Sabouraud dextrose agar incubated at 25-30°C for up to several weeks.^13^

The treatment for systemic sporotrichosis involves 2 weeks of amphotericin B at 0.7-1.0 mg/kg once daily, followed by oral antifungal therapy such as itraconazole, administered at 200 mg twice daily for 1 year. This regimen has a success rate of 60%-80%.^10,14^ Routine surgical debridement is not generally supported in the literature; however, in advanced cases, surgical debridement, synovectomy and even arthrodesis can be considered as adjuncts to antifungal therapy.^15^ In our case, the patient underwent adjunct surgical debridement and went on to complete the treatment regimen, resulting in good recovery.

Our case emphasises the importance of completing the prescribed regimen and strictly adhering to medication, with multidisciplinary involvement, to ensure the complete eradication of the infection and improve patient outcomes.

## Conclusion

Disseminated extracutaneous sporotrichosis with multifocal bone involvement can occur in patients with uncontrolled diabetes mellitus and poor adherence to antifungal treatment. A biopsy and fungal culture are tools to confirm the diagnosis. Applying a multidisciplinary approach, debriding the infected bone and ensuring long-term adherence to medication are key strategies in patient recovery.
